# Cyber Offending Among Young People: an Analysis of the Correlates for Different Categories of Cyber-Dependent Offending Using the Triple Risk Delinquency Framework

**DOI:** 10.1007/s40865-026-00317-9

**Published:** 2026-08-08

**Authors:** Marleen Weulen Kranenbarg, Abel Gonzalez-Garcia

**Affiliations:** 1 Department of Criminology, Vrije Universiteit Amsterdam, Amsterdam, Netherlands; 2 Universidad a Distancia de Madrid, Madrid, Spain

**Keywords:** Cyber-offending, Triple risk delinquency model, Cyber-dependent crime, Individual risk factors, Social risk factors, Opportunity risk factors

## Abstract

Cyber-dependent offenses involve the exploitation of information technologies to target digital infrastructures, such as hacking, malware, and distributed denial-of-service (DDoS) attacks. This study applied the Triple Risk Delinquency (TRD) framework as an analytical lens to compare personal, social, and situational or opportunity risks between distinct categories of cyber-dependent offending. We drew on data from a sample of 725 Dutch students aged 12–25 (*M*_*age*_=16.62 years; *SD* = 1.99; 25.66% female) who were following IT programs, tracks, or courses in secondary or tertiary education. To address gaps in the existing literature on cyber-dependent crime, we first identified distinct subcategories of cyber-dependent offending using principal component analysis: (1) technical hacking, (2) using or creating technical tools, and (3) misuse of unauthorized access. Second, we aimed to identify and classify correlates associated with these categories, utilizing the TRD model as an organizing framework. Logistic regression models indicated that prior victimization and peer offending are consistently positively associated with all categories of cyber-dependent crime, underscoring their general significance as personal and social correlates. Offence-specific patterns emerged as well: higher IT knowledge was associated with technical hacking, whereas lower self-control was associated with misuse of unauthorized access. Opportunity risks (such as above-average risky online activities) were more salient for technical hacking and tool use than for misuse of access, and stronger parental rules related negatively to technical hacking. The differences in correlates between these three categories underscore the need for future research that differentiates between different types of cyber-dependent crime.

## Introduction

In discussions concerning cyber-dependent offenses among young people, scholars such as Yar (2005), Rokven et al. (2018), and Loggen et al. (2024) have emphasized that criminology, and more specifically developmental criminology, has paid insufficient attention to this specific form of deviance, despite its increasing relevance to policymakers, law enforcement authorities, and the media in recent years. Although preventative measures have emerged in response to this issue, they are frequently grounded in anecdotal observations and experiential practice, rather than robust empirical and theoretical frameworks (see, for example, an inventory of Dutch interventions in the field of youth and cybercrime by CCV (2023). This highlights the urgent need to examine the specific correlates of cyber-dependent offending among young people.

More specifically, while some studies have examined risk and protective factors associated with cyber-dependent behaviors among young people through frameworks like the General Theory of Crime (Back et al., 2018; Holt et al., 2012, 2021; Marcum et al., 2014) or social learning theory (Fox & Holt, 2021; Holt et al., 2010), there is a lack of research employing integrative theoretical models that combine risk domains to understand offending, such as the Triple Risk Model for Delinquency developed by Redondo (2008, 2015).

Furthermore, scholarly inquiry into distinct subtypes or classifications within the large category of cyber-dependent crime remains limited, particularly in relation to correlates and life-course criminological theory (Loggen et al., 2024; Weulen Kranenbarg et al., 2022). Cyber-dependent offenses constitute technologically mediated crimes where information and communication technologies are both the tools and targets of illegal activity, including hacking, malware deployment, and distributed denial-of-service (DDoS) attacks (see Phillips et al. (2022) for an overview of definitions and classifications of cybercrime). Often, these offenses are studied alongside cyber-enabled behaviors (e.g., cyberbullying, sexting, and online dating abuse), under the broader umbrella of general cyber deviance (Virgara & Whitten, 2023; Holt et al., 2012). While some scholars distinguish between cyber-enabled and cyber-dependent offending in their analyses (e.g., Rokven et al., 2018; Weulen Kranenbarg et al., 2022), the latter remains a broad category that includes both rudimentary and sophisticated forms of offending, ranging from simple password guessing to advanced malware use. Other studies, by contrast, isolate one specific offense type such as hacking (Fox & Holt, 2021; Holt et al., 2010), limiting comparative insight into differing correlates.

Below we first introduce the Triple Risk Delinquency framework. Afterward, we use this framework as our organizing lens to discuss the limited literature on correlates of cyber-dependent offending among young people. Subsequently we discuss the aims of our study, followed by the methods section that details our methodological and analytical approach. In this section we also discuss which distinct subcategories of cyber-dependent offending we identified in the data. In the results section we first discuss individual correlates of each subcategory, followed by a comparison of correlates between subcategories. In the conclusion and discussion section we first describe and interpret our findings and afterwards discuss the theoretical implications of these findings. Finally, we discuss the limitations of our study and how our this explorative application of the TRD framework could lead to more detailed tests in future research.

## The Triple Risk Model for Delinquency (TRD model): an Integrative Framework

In response to a general need in Criminology for integrative models capable of organizing and explaining the multifaceted processes related to offending, the Triple Risk Model for Delinquency (TRD model), developed by Redondo (2008, 2015), offers a comprehensive theoretical framework that unifies diverse criminological perspectives. Drawing upon the foundations of developmental and life course criminology, the TRD model posits that delinquency arises from a combination of three categories of risk: personal, social, and opportunity (see also Bobbio et al., 2021).

Personal risks refer to individual characteristics and early developmental factors that impair normative socialization processes. These may include impulsivity, low empathy, poor emotional regulation, cognitive deficits, neuropsychological vulnerabilities, or early behavioral problems. Such traits often make it difficult for individuals to internalize social norms and develop self-control (Redondo, 2008, 2015).

Social risks involve deficits in the socialization process due to a lack of adequate support systems. These risks are rooted in the quality of relationships with family members, peers, schools, and other social institutions. For example, inconsistent parenting, family conflict, associating with delinquent peers, or school disengagement can all create environments where antisocial behavior is more likely to flourish (Redondo, 2008, 2015).

Opportunity (or situational) risks encompass the contexts or stimuli that provide concrete opportunities for offending. These risks are not necessarily linked to long-term development but relate to the immediate environment that facilitates the commission of a criminal act, such as lack of guardianship, availability of targets, or peer pressure in a specific moment (Redondo, 2008, 2015).

What distinguishes the TRD model is its emphasis on the combination of these categories (Bobbio et al., 2021). According to Redondo (2008, 2015), delinquent behavior is most likely to occur when personal and social risk factors combine to generate a criminogenic disposition or motivation, which is then actualized through situational opportunities. Note, however, that given the explorative nature of the present study the TRD model serves primarily as an analytical framework to organize risk domains across offence categories.

By combining personal, social, and opportunity risks within a single explanatory structure, the TRD model provides a nuanced understanding of the pathways to delinquency. It not only identifies key mechanisms involved in the emergence of deviant behavior, but also offers a framework for designing targeted interventions. Prevention and treatment programs inspired by this model would ideally address multiple domains simultaneously—for example, strengthening family support while enhancing cognitive-behavioral skills and reducing exposure to criminogenic environments (Bobbio et al., 2021; Redondo, 2008, 2015).

In sum, the TRD model complements and advances the goals of developmental and life course criminology by offering an integrative explanation of offending that is both theoretically robust and practically applicable (Bobbio et al., 2021; Redondo, 2008, 2015). Its holistic perspective is particularly suited to guide research and intervention efforts aimed at addressing cyber-dependent offenses and other emerging forms of delinquency. Since it’s development the TRD model has been used in some empirical studies on other forms of crime (Bobbio et al., 2021; González García, 2016; González García & Campoy Torrente, 2018; Pérez Ramírez, 2012) and in the development of a risk assessment tool (Redondo & Martínez, 2014).

## Correlates for Cyber-Dependent Offending Organized by TRD Categories

Despite the growing attention to cybercrime in criminological literature, only a limited number of quantitative studies have explicitly investigated cyber-dependent offending as a distinct outcome variable. This represents a significant gap, given the potentially unique personal, social, and opportunity risks of those involved in such offenses. In this section, we draw upon recent meta-analytic and systematic reviews to classify previously identified correlates for cyber-dependent offending using the TRD framework (Redondo, 2008, 2015). This classification is summarized in Table 1. In doing so, we explore whether a combination of personal risks, social risks, and opportunity risks can be found for cyber-dependent offending.

**Table 1:** TRD model classifications of correlates identified in reviews of previous research on cyber-dependent crime

Study	Cybercrime Type	Personal Risks	Social Risks	Opportunity Risks
Wissink et al. (2023)	Hacking	Low self-control, poor online/offline behavior, high computer preoccupation, low moral standards	Low school preoccupation, deviant peers	None
Loggen et al. (2024)	Hacking	Male, early onset (< 12), prior victimization, IT fascination, high education, internet addiction, high IT knowledge, neutralization techniques, low self-control, risk-taking, poor social skills, prior offending	Antisocial online peers, low school attachment, unemployed, high SES, negative parental supervision	Gaming, access to technology/internet
Loggen et al. (2024)	Malware	Autism spectrum traits, lower cognitive skills, high IT knowledge	Deviant peers	None
Weulen Kranenbarg et al. (2022)	Cyber-dependent (broad)	Age, computer addiction, IT knowledge, low self-control	Peer offending	None

A recent meta-analytic review by Wissink et al. (2023) specifically examined correlates for cybercrime, though the scope was limited to hacking—excluding other forms of cyber-dependent crime such as malware development or Distributed Denial-of-Service (DDoS) attacks. Their analysis included ten quantitative studies (Brewer et al. 2018; Donner et al. 2014; Holt et al. 2012; Hu et al. 2013; Lee 2018; Rogers et al., 2006a, b); Seigfried-Spellar et al. 2015; Seigfried-Spellar and Treadway 2014; Udris 2016; Xu et al. 2013) conducted in diverse international contexts (e.g., the United States, South Korea, China, Canada, Australia, and a multi-country study involving 31 nations).

The initial classification as shown in Table 1 suggests that, at least in the context of hacking, empirical studies have predominantly focused on personal and social correlates, while neglecting situational opportunities that could facilitate offending behavior. This lack of attention to opportunity risks is notable, given the strong emphasis placed on opportunity structures in traditional criminological theories like Routine Activity Theory (Cohen & Felson, 1979) or Rational Choice Theory (Clarke & Cornish, 1985; Cornish & Clarke, 2017).

A more expansive analysis is found in the systematic narrative literature review by Loggen et al. (2024), which examined 86 studies (both quantitative and qualitative) related to various forms of cyber-dependent crime—specifically hacking, malware development, and DDoS attacks. Their review identified 27 distinct correlates, though, as they noted, the bulk of the research remains focused on hacking, with fewer studies addressing malware and almost none systematically exploring DDoS offenses (as indicated in Table 1). The classifications displayed in Table 1 reinforce conclusions discussed above based on the meta-analyses, while also highlighting important nuances. For instance, deviant peers consistently emerge as a relevant social correlate across various types of cyber-dependent offending. Meanwhile, opportunity risks, though occasionally mentioned in the context of hacking (e.g., access to technology), are rarely investigated with the same rigor as personal or social risks. This suggests a need for broader empirical attention to the situational dynamics of cybercrime, as emphasized by the TRD framework.

In addition to the aforementioned reviews, we discuss the research by Weulen Kranenbarg et al. (2022) who conducted a study using the same data employed in the present research. Their study compared cyber-dependent crime (including acts such as hacking by guessing passwords) with cyber-enabled and traditional offline crime, and substantial differences were found. For cyber-dependent crime significant personal correlates were age, computer addiction, IT knowledge, and low self-control. Among these, strong IT skills emerged as a particularly distinguishing feature. Interestingly, aside from peer offending, no environmental factors (neither social nor opportunity risks) were found to be significantly related to cyber-dependent crime, even though such factors did show relevance in cyber-enabled and traditional offenses. Variables such as being home alone, satisfaction with IT education, or parental/school rules did not appear to influence cyber-dependent offending.

Although the reviews summarized above identify a broad range of personal, social, and opportunity risks, not all of these factors are available in our dataset. Therefore, in the current study we select, within each of the three TRD domains, those indicators that most closely match the constructs highlighted in previous research. For example, personal risks such as low self-control, IT knowledge, and signs of computer addiction were available in our data, whereas other factors (e.g., moral standards, Autism spectrum traits) were not included in the original survey. Similarly, among social and opportunity-related factors we include only those variables that conceptually fall within the TRD framework and were available in our dataset. This approach allows us to stay theoretically aligned with the TRD model while making empirically grounded use of the measures at hand.

In parallel to the broader literature reviewed above, our research group has conducted a series of studies on cyber-dependent offending among young people. Earlier work demonstrated that cyber-dependent offenders differ from traditional offenders in important ways and that cyber-dependent offending follows partly distinct developmental patterns (Weulen Kranenbarg et al., 2018). Subsequent research examined the role of peer networks and showed that cyber-dependent offenders are embedded in social environments in which cyber-offending behaviors may be learned and reinforced (Weulen Kranenbarg et al., 2021). More recently, research among a high-risk sample of students in IT-related education highlighted the importance of factors such as technical skills, peer offending, and computer-related behaviors in explaining cyber-dependent offending (Weulen Kranenbarg et al., 2022). Together, these studies have improved our understanding of who engages in cyber-dependent offending and under which circumstances. However, they largely treated cyber-dependent offending as a single category. The present study builds on this line of research by differentiating between categories of cyber-dependent offending and examining whether their correlates differ across the personal, social, and opportunity domains of the Triple Risk Delinquency framework.

### The Current Study

The current paper uses data from a large sample (N = 725; *M*_*age*_=16.62 years; *SD* = 1.99; range 12–25; 25.66% female) of Dutch students who were following IT programs, tracks, or courses in secondary or tertiary education. This specific sample is needed to examine behavior that requires certain technical skills that are not very prevalent in the general population. Previous research has also shown that students in IT-education can be considered at high-risk for committing cyber-dependent crime (Weulen Kranenbarg et al., 2018, 2022) and in our data, even if we exclude hacking by guessing a password (the most frequently reported type of cyber-dependent crime in this sample) which is not technically sophisticated, 36% of the respondents in this high-risk sample still self-report one or more forms of cyber-dependent offending (that are more sophisticated). With this data we aim to answer the following research question: *“How are factors related to personal risk*,* social risk*,* and opportunity risk differently related to specific categories of cyber-dependent crime?”*. The main objective of this research is to find out which correlates are present in young technical cyber-dependent offenders using the TRD model for the classification of these correlates. Based on self-reported cyber-dependent offending, we first identify different categories of the more technical types of cybercrime in our sample. By disaggregating offense types, we provide one of the first comparative assessments of risk patterns across specific forms of cyber-dependent crime. For each of the three resulting categories (technical hacking, using or creating technical tools, and misuse of unauthorized access) we estimate logistic regression models with explanatory variables across the types of risk in the TRD framework (personal, social, opportunity). By comparing the outcomes between the different types of crime, we show how a more detailed look at cyber-dependent crime reveals that some types of risk are specific to a specific type of crime, while other types of risk are more generally related to technical forms of cyber-dependent offending.

## Methods

### Sample and Procedure

For this paper we used data from the “*Understanding cybercriminal behaviour among young people*” project, funded by the UK Home Office. As justified in detail in the “Current Study” section above, this study was conducted among a high-risk sample of students in IT-related education (IT programs, tracks, or courses in secondary or tertiary education). Data was collected at eight tertiary schools, three higher level secondary schools, and one lower level secondary school. Schools were selected purposively based on whether they offered Computer Science or IT programs, ensuring that we recruited respondents with sufficient technical skills and elevated risk of cyber‑dependent offending. Using national open‑source educational data, we identified and invited secondary and tertiary schools with substantial IT enrolment within feasible travel distance. See for details about data collection Weulen Kranenbarg et al. (2022). All schools provided some form of Computer Science or IT program to their students, and we only selected students who were following these programs (either as an elective or as mandatory part of their program).

A total of 892 students participated in wave 1. Participation rates varied across schools, ranging from approximately 25% to 100%, although these figures should be considered estimates because participating schools could not provide complete lists of all eligible students. For the current study, we used data from the 725 respondents who also participated in wave 2 (*M*_*age*_ = 16.62 years; *SD* = 1.99; range 12–25; 25.66% female). Between wave 1 and wave 2, 167 respondents dropped out, resulting in a retention rate of 81.3% (attrition rate = 18.7%). Attrition was partly due to students switching educational programs or discontinuing their IT track. Because follow-up data collection was organized through the participating schools, students who had left the participating school before wave 2 were not re-contacted and were therefore not eligible for participation in the follow-up survey.

For both waves, respondents completed a survey in their classroom. Wave 1 took place between September and November 2019, and wave 2 was conducted between January and February 2020, meaning that the two waves were approximately three months apart. Prior to data collection, participating schools distributed digital study information and consent forms developed by the researchers. Students provided informed consent before participation. Similarly, parental consent was obtained for all respondents under the age of 16, in line with EU data protection regulations.

### Dependent Variables

For the dependent variables in this paper we use self-report data from wave 2. The original survey included items on cyber-dependent, cyber-enabled and traditional crime. For this paper we only selected the items on cyber-dependent crime and excluded the item on hacking by guessing a password, as we are specifically interested in more “technical” types of cybercrime that require at least some knowledge or skill in this area. Respondents reported whether they had engaged in each cyber-dependent offense during the three months preceding the wave 2 survey. For the present analyses, the individual offense items were coded dichotomously, indicating whether the respondent reported committing the offense at least once during that period. Table 2 shows prevalence rates of the items that were included in the analyses. Details on how individual items were measured can be found in Appendix 1.

**Table 2:** Prevalence rates of self-reported cyber-dependent crimes (*N* = 725)

	*N*	%
Hacking: technical applications	73	10.07
Hacking: exploits	80	11.03
Hacking: SQL injections	54	7.45
Hacking: other means	78	10.76
Illegally copying files or data	159	21.93
Vandalizing or modifying data	113	15.59
Defacing websites	64	8.83
DDoS with help of others	41	5.66
DDoS self	44	6.07
Using malware	33	4.55
Writing malware	39	5.38

To identify categories of crime, we used principal-component analysis with varimax rotation. We identified three factors with an eigenvalue above 1. Based on the highest factor loading in the rotated pattern matrix in Table 3 we assigned each item to one of the three categories. *Technical hacking* (*α* = 0.85) captures the use of technical methods to gain unauthorized access to computer systems. The category includes all items referring to technically mediated forms of hacking. Using or creating technical tools (*α* = 0.83) reflects involvement in the use or development of technical tools designed to disrupt or compromise computer systems. The category includes all DDoS- and malware-related items. Respondents who self-reported these crimes either used technical tools created by others to carry out DDoS or malware attacks, or they created (and used) these tools themselves. Misuse of unauthorized access (*α* = 0.73) reflects the exploitation of previously obtained unauthorized access (by any form of hacking) to a computer system. The category includes offenses involving the theft, modification, or destruction of digital data and website defacement. In our analyses we used dichotomous outcome variables for these three categories. Prevalence rates can be found in Table 3.

**Table 3:** Pattern matrix principal-component analyses and prevalence rates of resulting categories

	Factor		
	1	2	3
	Technical hacking	Technical tools	Misuse unauthorized access
Alpha	0.85	0.83	0.73
Prevalence rates N(%)	130(17.93)^1^	76(10.48)^2^	198(27.31)^3^
Hacking: technical applications	0.74	0.29	0.25
Hacking: exploits	0.79	0.26	0.15
Hacking: SQL injections	0.77	0.21	0.23
Hacking: other means	0.79	0.16	0.15
Illegally copying files or data	0.16	0.11	0.83
Vandalizing or modifying data	0.22	0.18	0.83
Defacing websites	0.50	0.23	0.51
DDoS with help of others	0.29	0.78	0.04
DDoS self	0.37	0.71	0.13
Using malware	0.07	0.81	0.23
Writing malware	0.26	0.75	0.19

NOTE: 1: 32(4.41%) missing; 2: 24(3.31%) missing; 3: 24(3.31%) missing

### Independent Variables

Following the literature review, we classified predictors available in our data into the three TRD domains: personal, social, and opportunity risks. Importantly, while previous reviews identify a wide range of potential correlates of cyber-dependent crime, our analyses rely on those measures that were available in our survey and that most closely reflect the constructs emphasized in prior work. The variables listed below should therefore be interpreted as domain-representative indicators rather than exhaustive measures of all TRD-based risks. Where possible we used independent variables from wave one to predict self-reported offending in wave two. However, many items were only included in wave two. Therefore, it is important to note that our analyses show relationships, but the data does not allow for any causal claims. Table 4 shows descriptive statistics of the independent variables (See Appendix 2 for the correlation matrix). Below we describe the meaning of each variable, followed by information on how the construct was operationalized. Details on how individual items were measured can be found in Weulen Kranenbarg et al. (2022).

**Table 4:** Descriptive statistics of independent variables (*N* = 725)

Continuous variables	Mean	SD	Range
Age	16.62	1.99	12–25
Computer addiction	1.37	0.39	1–3
IT-knowledge	3.23	1.34	0–6
Low self-control	2.90	0.65	1.33–4.89
Social competence online	3.50	0.93	1–5
Home alone, average day	1.23	1.30	0–5
IT education satisfaction	4.01	0.95	1–5
Talk computer activities	1.60	0.98	1–5
Rules parents	3.29	0.86	1–5
Rules school	3.29	0.81	1–5
Dichotomous variables	N	%	
Victimization	190	26.21	
Peer offending	202	27.86	
Risky online activities	416	57.38	

#### Personal risk 

*Age* was included as a demographic indicator and was measured in years at wave 1. *Computer addiction* refers to problematic and excessive computer use. Higher scores indicate stronger symptoms of addictive computer use. The construct was measured using a six-item scale based on the game addiction scale by Lemmens et al. (2015), covering preoccupation, toleration, persistence, escapism, deception, and problems (wave 1; *α* = 0.74). *IT-knowledge* reflects respondents’ objective technical knowledge and skills. This construct was measured using a five-item IT knowledge quiz (wave 2), with scores indicating the number of correctly answered questions. The questions were a selection of the IT-quiz questions developed by Weulen Kranenbarg et al. (2021). We included the questions that proved to be most suitable to distinguish between high and low-skilled respondents. *Low self-control* reflects impulsivity, risk-seeking, and difficulties with behavioral regulation. Higher scores indicate lower levels of self-control. The construct was measured using a composite scale based on Grasmick et al. (1993) (wave 2; *α* = 0.72). *Victimization* captures respondents’ cybercrime victimization. The dichotomous measure indicates whether respondents reported having been victimized between waves 1 and 2 through hacking, data misuse, malware, or game-related attacks involving hacking or DDoS techniques. *Social competence online* refers to respondents’ perceived ability to interact effectively in online environments. Higher scores indicate greater online social competence. The construct was measured using an adapted version of the social competence scale developed by Lemmens et al. (2011), focusing on online rather than offline interactions (wave 2; *α* = 0.81).

#### Social risk 

Home alone reflects the extent to which respondents spend time at home without adult supervision. The variable measures the number of hours respondents spend at home alone on an average weekday (wave 1). Answers ranged from 0 to 5, indicating the following categories 0 h; 1–2 h; 3–4 h; 5–6 h; 7–8 h; more than 8 h. *IT education satisfaction* reflects respondents’ perceived quality and challenge of their IT education. Higher scores indicate greater satisfaction. The construct was measured using two items assessing whether students felt challenged during IT classes and whether they believed they learned new IT skills (wave 1; *α* = 0.71). *Talk computer activities* reflects communication between students and teachers about students’ computer use outside school. Higher scores indicate more frequent discussion of these activities with teachers. *Peer offending* captures exposure to cyber-offending peers. The dichotomous measure indicates whether respondents reported that at least one friend (school, offline, or online) engaged in one or more of the cyber-dependent offenses included in this study.

#### Opportunity risk 

To measure opportunities, we included two variables about online and offline rules, from parents and at school. *Rules parents* reflects the extent to which parents monitor and regulate their children’s online and offline activities. Higher scores indicate stronger parental monitoring and rule-setting. The measure combines scales assessing offline rules (*α* = 0.77) and online rules (*α* = 0.88). These items were based on Bruinsma et al. (2015), Hoeben and Weerman (2016) and Janssen et al. (2017). Similarly, *rules school* reflects the extent to which schools communicate, monitor, and enforce behavioral rules in both online and offline settings. Higher scores indicate stricter and more consistently enforced rules. The measure combines scales assessing offline rules (*α* = 0.78) and online rules (*α* = 0.80). Thes scales were inspired by the work of Gordon (2018), Nagin (2013), and Zullig et al. (2010). *Risky online activities* captures engagement in online activities that may create opportunities for cyber-dependent offending. Based on previous research, we focused on gaming, forum participation, and programming activities. The dichotomous measure identifies respondents who spent relatively high amounts of time on at least one of these activities compared to the rest of the sample, namely one hour or more per weekday on forum participation or programming, or three or more hours per weekday on gaming.

### Analytical Strategy

As our three outcome variables are dichotomous, we use logistic regression models. For each outcome variable we first estimate separate models for variables representing the three different types of risk: personal risk, social risk, and opportunity risk. Lastly, we estimate a model including the variables from these three models that were statistically significant in one final model including all three types of risk.

## Results

For each category of offending we first ran separate models for personal risk, social risk, and opportunity risk (see Appendix 3). In these models, some of the included correlates were not statistically significant for one or more of the outcome categories. These correlates were: age, IT education satisfaction, and rules school. Non-significant variables were excluded from the final analyses. Table 5 shows the results of these final logistic regression models that include all three types of risk for the three crime categories. Below we first discuss the results for each crime category separately. Afterwards, we compare the similarities and differences between the models.

**Table 5:** Final logistic regression models for the three offending categories

	Technical hacking			Technical tools			Misuse unauthorized access		
	b	SE	OR	b	SE	OR	b	SE	OR
Personal risk									
Computer addiction	0.76**	0.27	2.14				0.57*	0.25	1.78
IT-knowledge	0.24*	0.10	1.27						
Low self-control							0.43**	0.15	1.54
Victimization	0.73**	0.23	2.09	1.00***	0.27	2.73	0.58**	0.20	1.79
Social competence online							0.16	0.11	1.18
Social risk									
Home alone, average day	0.14	0.08	1.15	0.21*	0.09	1.23	0.05	0.07	1.05
Talk computer activities	0.18	0.11	1.20						
Peer offending	0.78***	0.23	2.19	1.00***	0.27	2.71	0.89***	0.20	2.45
Opportunity risk									
Rules parents	−0.30*	0.14	0.74				−0.20	0.11	0.82
Risky online activities	1.02***	0.28	2.76	0.96**	0.32	2.60	0.33	0.20	1.39
Constant	−4.15***	0.75	0.02	−3.84***	0.34	0.02	−3.69***	0.74	0.02
Likelihood ratio chi-square			108.85			57.68			89.90
Pseudo R2			0.17			0.13			0.11
N			654			675			661

*** *p* <.001; ** *p* <.01; * *p* <.05

For personal risk we found that computer addiction, more IT-knowledge and victimization are all significant and positively related to technical hacking and these results hold in the final model. The pseudo R2 shows that Model 1 with only personal correlates explains 13% of the variance in technical hacking. In contrast, Model 2 with only social correlates, only explains 6% of the variance. While the time spent home alone without supervision on an average day, the extent to which students talk with teachers about their online activities, and peer offending are all significant and positively related to technical hacking in this model, only peer offending is still significant in the final model. Model 3, including only opportunity risks, explains 9% of the variance in technical hacking. The significant predictors in this model show that more parental control is negatively related to technical hacking, while more than average risky online activities are significantly positively related to technical hacking. These results hold in the final model. The final model, including personal, social, and opportunity risks, can explain 17% of the variance in technical hacking.

In contrast to technical hacking, there was only one personal risks that was significantly related to using technical tools: victimization. Nevertheless, this is a strong predictor and the personal risk model can still explain 8% of the variance in using technical tools. This variable is still significant in the final model. Similarly, social risks in Model 2 can explain 7% of the variance. The time spent home alone without supervision on an average day and peer offending are significant and positively related to using technical tools and these results hold in the final model. Again, for opportunity risks there was only one significant variable that showed that more than average risky online activities were significantly positively related to using technical tools. This model on opportunity risks still explains 5% of the variance and the results hold in the final model. Compared to the model on technical hacking, the final model on using technical tools explains less variance, but still 13%.

For misuse of unauthorized access, we found again more significant personal risk predictors, which explain 8% of the variance in misuse of unauthorized access. Computer addiction, low self-control, victimization and online social competences are all significant and positively related to this category of behavior. However, the variable on social competence is not significant anymore in the final model where we control for other risks as well. Similar to using technical tools, we found that the time spent home alone without supervision on an average day and peer offending were significant and positively related to misuse of unauthorized access. This model on social risks explains 6% of the variance in this category of behavior. However, the effect of the time spent home alone without supervision is not significant anymore in the final model. In contrast to the other two crime categories, opportunity risks appeared less important for misuse of unauthorized access. This model only explains 3% of the variance. While we found a significant negative effect of parental supervision and a significant positive effect of risky online activities in Model 3, these results did not hold in the final model and there were no significant opportunity risks in the final model on misuse of unauthorized access. This is also reflected by the explained variance in the final model, only 11%, as this is the lowest compared to the other models on technical hacking and technical tools.

## Comparison Between Crime Categories

In Table 6 we compare the final models between the three crime categories. Two variables emerged as strong predictors for all crime categories: victimization and peer offending. On the other hand, some risks appeared to be specific for specific crime categories. With respect to personal risk, IT-knowledge was only related to technical hacking, whereas low self-control was only related to misuse of unauthorized access. With respect to social risk, the time spent home alone without supervision on an average day is only related to using technical tools. Finally, with respect to opportunity risk, it is interesting to see that the rules by parents are only related to technical hacking. Additionally, none of the opportunity risks included were related to misuse of unauthorized access. Finally, there was some overlap in the risks related to technical hacking and one of the other two categories. Computer addiction was related to both technical hacking and misuse of unauthorized access, while more than average risky online activities were related to both technical hacking and using technical tools.

**Table 6:** Comparison of significant variables (in the final model) between crime categories

	Technical hacking	Technical tools	Misuse unauthorized access
Personal risk	+ Victimization + Computer addiction + IT-knowledge	+ Victimization	+ Victimization + Computer addiction + Low self-control
Social risk	+ Peer offending	+ Peer offending + Home alone, average day	+ Peer offending
Opportunity risk	- Rules parents + Risky online activities	+ Risky online activities	
Pseudo R2	*0.17*	*0.13*	*0.11*

When comparing the pseudo R2 for these models, it is clear that the risks included in these analyses are more suitable to explain technical hacking. For both using technical tools and misuse of unauthorized access, we found that two of the risk categories only included one (or no) significant variable and the pseudo R2 for these models was also lower. For all crime types, the models on personal risk perform best in explaining the variance in offending. However, after personal risks, social risks are more important for using technical tools and misuse of unauthorized access, while opportunity risks are more important for technical hacking.

## Discussion and Conclusion

In this paper we used data from a large high-risk sample (*N* = 725) of students in IT-related education to analyze cyber-dependent offending by using the Triple Risk model for Delinquency (Redondo, 2008, 2015). We analyzed how personal risk, social risk, and opportunity risk differently related to specific categories of cyber-dependent crime. Based on self-reported cyber-dependent offending, we first identified three different categories of the more technical types of cyber-dependent crime in our sample: technical hacking, using or creating technical tools, and misuse of unauthorized access. For each category we estimated logistic regression models for the types of risk in the triple risk model. When comparing the outcomes of these models for the different categories, we found clear support for differentiating between different types of cyber-dependent crime and stepping away from the common use of broader categories (as also suggested by Loggen et al. (2024). By comparing the models of the different types of crime, we showed how a more detailed look at cyber-dependent crime revealed that some types of risk were specific to a specific type of crime, while other types of risk were more generally related to technical forms of cyber-dependent offending.

For personal and social risk we found two variables that were consistently related to all categories of cyber-dependent crime: victimization and peer-offending. Interestingly, these are both factors that are also consistently related to many types of traditional offending. First, for personal risk, we found that victimization is positively related to all types of cyber-dependent crime, which is consistent with limited previous research, although this correlate is often neglected in cybercrime studies (Loggen et al., 2024; Maras & Greenwood, 2024; Rokven et al., 2018; Wissink et al., 2023). For cybercrime specifically, there is some anecdotal evidence that some young people who are involved in cybercrime were first victimized, for example in games (Leukfeldt & Bekkers, 2026; Loggen et al., 2024; Maras & Greenwood, 2024). If they are DDoSed or hacked by their opponent, they will try to use those techniques as well, in the game and later on also in other situations. The other way around, victimization might also be a consequence of their own offending, for example when their opponents are taking revenge and hacking or DDoSing them back. As we measured victimization in the same wave as offending (wave 2), we do not know to what extent this is a causal relationship and what comes first. Second, based on previous research on cybercrime (Loggen et al., 2024; Wissink et al., 2023), it is no surprise that the social risk of peer-offending is also consistently related to all types of cyber-dependent crime. While the general news media might sometimes depict technically skilled cyber-offenders as loners, committing the crimes on their own from their own bedroom, our research shows that at least in our high-risk sample there was an important social aspect to these offenses as well.

When looking at differences between the models we found that the personal risk of computer addiction was not related to the use of technical tools, while it was related to both technical hacking and the misuse of unauthorized access. This might be related to the goal of the offense and how an offender is trying to reach that goal. Technical forms of hacking and subsequent misuse of unauthorized access might require a lot of time-investment. For some offenders, learning from the trial-and-error process of obtaining that unauthorized access might even be their main goal (Steinmetz, 2015). This suggests that, for some young people, sustained engagement in computer use may function as a context in which technical skills and offending opportunities gradually converge. Investing in the skills that are required for these activities and spending the time necessary to acquire unauthorized access might result in problematic addictive computer behavior. The use of technical tools such as malware or DDoS attacks might be a more quick solution and is more clearly linked to a specific goal. Therefore, offenders using these techniques might not display addictive computer behavior.

In a similar vein, more IT-knowledge was only related to technical hacking. This suggested that this was the most advanced type of offending in our dataset. Even within this high-risk group of offenders there was a subgroup that had the strongest skills and was committing these most advanced offenses. This pattern may indicate that as technical proficiency increases, the range of possible offending opportunities also expands, highlighting a potential developmental pathway shaped by continuous skill acquisition. Interestingly, while low self-control has often been linked to crime, also to cyber-dependent crime (Loggen et al., 2024; Wissink et al., 2023), our results showed that it was only related to the misuse of unauthorized access, not to the use of technical tools or technical hacking. In contrast to technical forms of hacking, unauthorized access might be gained fairly easily by, for example, guessing a password. This effect of low self-control might show that for misusing unauthorized access a person generally does not need strong technical skills. Additionally, even if a person used technical ways to gain unauthorized access for the purpose of learning from that process, having low self-control might be the reason why that person is not able to stop once they are in. After all that effort, having access to a computer system and all the data and capabilities of that system might be simply too tempting for someone with low self-control (Steinmetz, 2015) even if they had good intentions when they started hacking (Weulen Kranenbarg et al., 2018).

Apart from the general social risk of peer support, we found only one other significant social correlate which is only related to the use of technical tools, that is being at home alone without supervision. The absence of supervision is, apparently, not related to hacking or the misuse of unauthorized access. On the other hand, with respect to opportunity risk, we found that stronger parental rules that are restricting opportunities were negatively related to technical hacking. This indicates that opportunity structures in the home environment may influence not only whether young people offend, but also which types of offences feel accessible or feasible to them. This suggested that, for both more technical types of offending in our data, informal social control (either by the presence of parents or by their rules and restrictions that limit opportunities) was related to less offending. For misuse of unauthorized access, on the other hand, the absence of such an effect, combined with the positive link to peer offending and low self-control, might show that these offenders are seizing an opportunity created by unauthorized access without considering the negative reactions from their parents. Again, this suggested that these types of behavior are different and should not be combined in one outcome variable.

Finally, we found again more similarities between technical hacking and the use of technical tools with respect to opportunity risks based on risky online activities. These online activities (gaming, forum use, and programming) provide specific opportunities that are linked to these offenses. Engaging intensively in such online spaces may also increase exposure to norms, techniques, or challenges that subtly shape how young people perceive the boundaries between legitimate exploration and offending. A person can find opportunities and motivations for hacking and using malware or DDoS attacks in games (although research in this area is rather mixed see Bekkers et al., 2025), find the required knowledge for these attacks on forums, and use programming to create opportunities for themselves. Opportunities for the misuse of unauthorized access, on the other hand, might be more varied and can arise in many situations. This might also be the reason why our model on the misuse of unauthorized access has the lowest pseudo R2, showing that it is more difficult to explain the variance in this type of offense with the personal, social, and opportunity risks included in this study.

An important consideration when interpreting these findings is that, although the TRD model distinguishes a broad range of personal, social, and opportunity risks, only a subset of these constructs was available in our dataset. As outlined in the literature review, the empirical cybercrime literature identifies additional factors such as moral standards, autism spectrum traits, or specific situational opportunity structures that were not part of the original survey. The indicators used in this study should therefore be seen as domain representative rather than exhaustive. This means that our findings primarily speak to the TRD aligned risk dimensions that could be operationalized and measured with the available data, and that additional, unmeasured risks may further differentiate cyber offending subtypes in future research.

## Theoretical Implications

The present study offers an empirical application of the Triple Risk Model for Delinquency (TRD; Redondo, 2008, 2015). Using a high-risk sample of young respondents enrolled in IT-related education, we examined three categories of cyber-dependent offending: (1) technical hacking, (2) use of technical tools (e.g., DDoS attacks, malware), and (3) misuse of unauthorized access. Across all three forms, our results confirmed that personal, social, and opportunity-related risks should not be studied in isolation, as these could all contribute delinquent behavior.

Our results also provided an example of the TRD principle of intra-risk reinforcement, as different correlates within one type of risk are relevant for a category of offending. For personal risk, for example, individuals engaged in technical hacking frequently reported prior victimization, higher levels of computer addiction, and more IT-skills. Similarly, misuse of unauthorized access was related to victimization, low self-control, and computer addiction — highlighting that multiple personal correlates can be related to this type of behavior. These findings echo those of González García (2016; González García & Campoy Torrente, 2018), who applied the TRD framework to the domain of cyberbullying. In a sample of almost 300 secondary school students, the study identified low self-esteem and high impulsivity as the key personal predictors of this type of online aggression. Additionally, lack of prosocial support (e.g., from teachers or parents) – social risks – and situational facilitators (such as anonymity and frequency of internet use) – opportunity risks – played significant roles.

In our study, social risk—particularly peer offending—emerged as a robust and consistent correlate across all offense types, validating its centrality within the TRD framework. Interestingly, the role of the social context varied by offense type. The use of technical tools was significantly associated with unsupervised time at home, potentially underscoring the criminogenic potential of isolated digital environments. These results align with findings from Loggen et al., 2024), who emphasized the role of (online) peer networks and subcultural norms in fostering cyber-offending.

The opportunity-related risks that we found further support the TRD model’s principle of integrating this type of risk. For technical hacking, more than average risky online activities (e.g., gaming, forum use, and programming) and the absence of parental rules were related to offending. This reinforced the idea that cyberspace affords not only access but also unregulated opportunity. However, in contrast, opportunity correlates did not retain significance in the final model predicting misuse of unauthorized access, suggesting that some cybercrimes may arise more spontaneously, or under different circumstances.

The current study is the first to apply the TRD-model to categories of cyber-dependent offending. Therefore, we used more basic models that provided insights into the different correlates, in the three categories, that can be relevant for these types of offending. As a consequence, it was beyond the scope of this paper to test different interaction effects of correlates within and between the different categories of risk. However, given the slight changes between the separate models for each category of risks and the final models including all categories, our results indicate that such interactions can further explain this type of offending, underscoring the principles of intra and inter-risk interaction which assume that different correlates can reinforce each other. Future research could, for example, focus even more specifically on one category of offending and study such interactions in more detail.

Beyond this convergence and interaction, this study reaffirms the TRD’s meta-theoretical aim of integrating core constructs from major criminological theories. Low self-control, a central concept in the General Theory of Crime, significantly predicted misuse of unauthorized access. Social Learning Theory is reflected in the role of deviant peer association for all categories of crime, and Routine Activity Theory is reflected in the effects of opportunity-related variables like lack of parental rules and risky online activities.

In sum, this research confirms that the TRD model is a useful framework in the field of cyber-dependent crime. By classifying and analyzing distinct types of technical offenses, we demonstrate that correlates do not operate in isolation, should be studied in combination, and should include opportunity risks. The differentiation among offense types further suggests that prevention and intervention efforts should be tailored, recognizing that different configurations of risk require targeted responses. Future research should continue to develop the TRD framework in digital contexts, particularly by incorporating more precise indicators of opportunity and further disaggregating cybercrime subtypes. Additionally, it is important to note that the present study used the TRD model primarily as an analytical framework to organize risk domains across offence categories, rather than as a full test of its core assumption that risks interact and reinforce one another. Future research should therefore examine combinations and interactions of personal, social, and opportunity risks more explicitly, as these dynamics are central to the TRD model and may reveal patterns that cannot be detected when correlates are modelled independently. This will allow for a deeper understanding of how risk factors evolve and interact in the digital lives of young people.

## Limitations and Future Research

The main limitation of this study lies in the measurement and operationalization of opportunity correlates. While personal and social risks were comprehensively assessed through validated instruments and multiple indicators, opportunity risks were more narrowly captured—primarily through indirect measures such as self-reported risky online activities and perceived parental or school rules. This limitation affects the explanatory depth for certain behaviors, especially misuse of unauthorized access, where no opportunity variables were statistically significant in the final model. Future studies should incorporate more nuanced and behavior-specific indicators of opportunity, including the quality and nature of online environments, peer exposure to criminal techniques, and real-time access to vulnerable systems or software. Additionally, qualitative research or ethnographic studies could provide richer insight into how young people perceive and seize online opportunities for offending, which often remain hidden in quantitative designs.

Another important limitation is the reliance on self-report data. Although this is common and useful in cybercrime research, it may lead to underreporting or biased recall, particularly in sensitive areas such as malware development or system intrusion. Further triangulation with observational data, school records, or digital behavior logs could strengthen and deepen future analyses.

Future research should also move beyond cross-sectional approaches and employ longitudinal designs to better capture the dynamic interplay of risk factors over time. By tracing how personal, social, and opportunity risks develop and interact across different stages of adolescence and young adulthood, scholars can better test the TRD model’s assumptions and potentially improve its predictive capabilities.

Furthermore, future research should focus more explicitly on developmental differences within cyber dependent offending. Our study included respondents spanning adolescence to emerging adulthood, all enrolled in IT related education and therefore still in a phase of skill development relevant to cyber offending. However, developmental criminology suggests that the importance of personal, social, and opportunity related risk factors can shift across adolescence and early adulthood. Studies with larger samples for specific age groups would allow for a more detailed examination of age specific manifestations of TRD related risks. Such research could identify whether risk configurations differ across developmental stages and could inform more targeted prevention strategies.

From a theoretical standpoint, future work should explore how the TRD model can integrate or differentiate cyber-dependent offending from traditional and cyber-enabled forms of delinquency. There may be shared or unique configurations of risk among these forms, and comparative analyses could refine the TRD model’s scope and applicability. Furthermore, interdisciplinary approaches that incorporate psychological, technological, and educational explanatory factors may yield a more holistic view of cyber-offending among youth.

Finally, there are some implications for prevention. The results highlight the need to account for heterogeneity among offenders: different subgroups of technical cyber-offenders show distinct configurations of correlates. This calls for targeted prevention strategies, rather than one-size-fits-all approaches. For instance, those with strong IT-skills and prior victimization may benefit more from personal interventions and programs directed at the positive use of IT-skills, while those embedded in peer networks of offenders may require social-based interventions and mentoring. Opportunity reduction strategies—such as parental supervision of online activities or digital literacy education—could also be strengthened, especially for those engaging in technical hacking. By using the TRD model as a guiding framework, prevention efforts can be multidimensional, adaptive, and more effectively tailored to the profiles and pathways of different types of cyber-offenders.

## Data Availability

No datasets were generated or analysed during the current study.

## Appendix 1. Measures

**Table:** Measures for self-reported cyber-dependent crimes

Short description	Question How many times since the last questionnaire did you without permission…
Hacking: technical applications	… hack through technical applications that help you guess passwords automatically? For example rainbow tables, brute force, or a key logger.
Hacking: exploits	… hack through exploits? These are programs or a piece of computer code with which you can use vulnerabilities in software.
Hacking: SQL injections	… hack through SQL injections? With an SQL injection you can read, modify, or even delete databases.
Hacking: other means	… hack through a way that hasn’t been mentioned in the previous questions about hacking? *
Illegally copying files or data	… copy digital files or data belonging to someone else?
Vandalizing or modifying data	… modify, delete, or add something to another person’s digital files or data?
Defacing websites	… alter the content of a web page, so that, for example, a different message on this website was displayed than the owners of the website intended? This is also called web defacement. (We’re not talking about putting “funny” texts on your friends’ social media accounts).
DDoS with help of others	… carry out a (D)DoS attack WITHOUT having set it up yourself? For example, ordered one over the internet, or had a friend do it for you.
DDoS self	… carry out a (D)DoS attack that you had (partly) set up yourself?
Using malware	… deliberately spread or use some form of malware?
Writing malware	… (partly) design or develop a form of malware?

* Given the focus of this paper we excluded the previous question on guessing a password (“… hack by guessing, peeking or filling in someone’s password yourself?”). This means that the “other” category mentioned here refers to other technical means of hacking

## Appendix 2

**Table:** Correlation matrix of independent variables

	1	2	3	4	5	6	7	8	9	10	11	12	13
1. Age	-												
2. Computer addiction	0.02	-											
3. IT-knowledge	0.23	0.08	-										
4. Low self-control	−0.07	0.22	−0.05	-									
5. Victimization	0.01	0.25	0.03	0.13	-								
6. Social competence online	0.02	0.11	0.02	0.15	0.09	-							
7. Home alone, average day	0.12	0.05	−0.06	0.11	0.05	0.07	-						
8. IT education satisfaction	−0.07	0.01	0.03	−0.09	0.00	−0.08	−0.08	-					
9. Talk computer activities	−0.03	0.06	0.10	0.08	0.06	0.05	0.03	0.11	-				
10. Peer offending	0.01	0.13	0.13	0.10	0.18	0.09	0.01	0.00	0.08	-			
11. Rules parents	−0.27	−0.06	−0.12	−0.10	−0.12	−0.08	−0.21	0.10	0.23	−0.14	-		
12. Rules school	−0.13	0.00	−0.02	0.03	0.03	−0.01	−0.05	0.14	0.17	0.02	0.23	-	
13. Risky online activities	0.22	0.22	0.22	0.06	0.18	0.16	0.07	−0.06	0.11	0.09	−0.15	−0.04	-

## Appendix 3

Separate logistic regression models on personal risk, social risk, and opportunity risk for each category of offending.

**Table:** a. Logistic regression models for Technical hacking

	Model 1			Model 2			Model 3			Model 4		
	b	SE	OR	b	SE	OR	b	SE	OR	b	SE	OR
Personal risk												
Age	0.07	0.05	1.07									
Computer addiction	0.91***	0.26	2.49							0.76**	0.27	2.14
IT-knowledge	0.40***	0.10	1.50							0.24*	0.10	1.27
Low self-control	0.24	0.17	1.27									
Victimization	1.03***	0.22	2.81							0.73**	0.23	2.09
Social competence online	0.21	0.12	1.23									
Social risk												
Home alone, average day				0.20**	0.08	1.22				0.14	0.08	1.15
IT education satisfaction				−0.16	0.11	0.85						
Talk computer activities				0.21*	0.10	1.23				0.18	0.11	1.20
Peer offending				1.06***	0.21	2.90				0.78***	0.23	2.19
Opportunity risk												
Rules parents							−0.39***	0.12	0.68	−0.30*	0.14	0.74
Rules school							0.15	0.13	1.16			
Risky online activities							1.46***	0.25	4.29	1.02***	0.28	2.76
Constant	−7.12***	1.16	0.00	−1.74***	0.47	0.18	−1.73**	0.55	0.18	−4.15***	0.75	0.02
Likelihood ratio chi-square			81.20			39.58			56.84			108.85
Pseudo R2			0.13			0.06			0.09			0.17
N			669			611			688			654

*** *p* <.001; ** *p* <.01; **p* <.05

**Table:** b. Logistic regression models for Technical tools

	Model 1			Model 2			Model 3			Model 4		
	b	SE	OR	b	SE	OR	b	SE	OR	b	SE	OR
Personal risk												
Age	−0.04	0.07	0.96									
Computer addiction	0.55	0.31	1.74									
IT-knowledge	0.14	0.11	1.15									
Low self-control	0.03	0.20	1.03									
Victimization	1.20***	0.27	3.31							1.00***	0.27	2.73
Social competence online	0.21	0.15	1.23									
Social risk												
Home alone, average day				0.21*	0.09	1.24				0.21*	0.09	1.23
IT education satisfaction				−0.20	0.13	0.82						
Talk computer activities				0.07	0.12	1.07						
Peer offending				1.22***	0.26	3.38				1.00***	0.27	2.71
Opportunity risk												
Rules parents							−0.23	0.15	0.80			
Rules school							−0.10	0.15	0.90			
Risky online activities							1.14***	0.30	3.14	0.96**	0.32	2.60
Constant	−4.09**	1.37	0.02	−2.14***	0.56	0.12	−1.82**	0.65	0.16	−3.84***	0.34	0.02
Likelihood ratio chi-square			36.20			30.78			23.10			57.68
Pseudo R2			0.08			0.07			0.05			0.13
N			676			619			697			675

*** *p* <.001; ** *p* <.01; **p* <.05

**Table:** c. Logistic regression models for Misuse unauthorized access

	Model 1			Model 2			Model 3			Model 4		
	b	SE	OR	b	SE	OR	b	SE	OR	b	SE	OR
Personal risk												
Age	−0.03	0.05	0.97									
Computer addiction	0.65**	0.23	1.91							0.57*	0.25	1.78
IT-knowledge	0.10	0.07	1.11									
Low self-control	0.47***	0.15	1.59							0.43**	0.15	1.54
Victimization	0.82***	0.19	2.27							0.58**	0.20	1.79
Social competence online	0.21*	0.10	1.24							0.16	0.11	1.18
Social risk												
Home alone, average day				0.15*	0.07	1.16				0.05	0.07	1.05
IT education satisfaction				−0.16	0.10	0.85						
Talk computer activities				0.17	0.09	1.19						
Peer offending				1.03***	0.19	2.79				0.89***	0.20	2.45
Opportunity risk												
Rules parents							−0.31**	0.10	0.73	−0.20	0.11	0.82
Rules school							−0.04	0.11	0.97			
Risky online activities							0.59***	0.18	1.81	0.33	0.20	1.39
Constant	−4.07***	0.97	0.02	−1.05*	0.41	0.35	−0.17	0.46	0.84	−3.69***	0.74	0.02
Likelihood ratio chi-square			64.66			42.10			24.91			89.90
Pseudo R2			0.08			0.06			0.03			0.11
N			674			619			696			661

*** *p* <.001; ** *p* <.01; **p* <.05

## References

[CR1] Back, S., Soor, S., & LaPrade, J. (2018). Juvenile Hackers: An empirical test of self-control theory and social bonding theory. *International Journal of Cybersecurity Intelligence & Cybercrime*, *1*(1), 40–55. 10.52306/01010518VMDC9371

[CR2] Bekkers, L., Holt, T. J., & Leukfeldt, E. R. (2025). Online gaming as a criminological environment: Exploring criminogenic needs and offending behaviors of gamers. *Journal of Criminal Psychology*. 10.1108/JCP-03-2025-0035. https://doi-org.vu-nl.idm.oclc

[CR3] Bobbio, A., Arbach, K., & Redondo, S. (2021). El Modelo del Triple Riesgo Delictivo en la explicación de la conducta antisocial de adolescentes varones y mujeres. *Revista Española De Investigación Criminológica*, *19*(1), 1–35. 10.46381/reic.v19i1.479

[CR4] Brewer, R., Cale, J., Goldsmith, A., & Holt, T. (2018). Young People, the Internet, and Emerging Pathways into Criminality: A Study of Australian Adolescents. *International Journal of Cyber Criminology*, *12*(1), 115–132. 10.5281/zenodo.1467853

[CR5] Bruinsma, G. J. N., Pauwels, L. J., Weerman, F. M., & Bernasco, W. (2015). Situational action theory: Cross-sectional and cross-lagged tests of its core propositions. *Canadian journal of criminology and criminal justice*, *57*(3), 363–398.

[CR6] CCV (2023). *Interventies op het gebied van jeugd en cybercrime. Aanzet voor de City Deal innovatie*. Centrum voor Criminaliteitspreventie en Veiligheid (CCV). https://hetccv.nl/app/uploads/2023/12/Interventies-op-het-gebied-van-Jeugd-en-Cybercrime-DEF-20231212.pdf

[CR7] Clarke, R. V., & Cornish, D. B. (1985). Modeling Offenders’ Decisions: A Framework for Research and Policy. *Crime & Justice*, *6*, 147–186.

[CR8] Cohen, L. E., & Felson, M. (1979). Social Change and Crime Rate Trends: A Routine Activity Approach. *American Sociological Review*, *44*, 588–608.

[CR9] Cornish, D. B., & Clarke, R. V. (2017). The rational choice perspective. In R. Wortley & M. Townsley (Eds.), *Environmental Criminology and Crime Analysis* (2nd ed., pp. 29–61). Routledge.

[CR10] Donner, C. M., Marcum, C. D., Jennings, W. G., Higgins, G. E., & Banfield, J. (2014). Low self-control and cybercrime: Exploring the utility of the general theory of crime beyond digital piracy. *Computers in Human Behavior*, *34*, 165–172.

[CR11] Fox, B., & Holt, T. J. (2021). Use of a multitheoretic model to understand and classify juvenile computer hacking behavior. *Criminal Justice and Behavior*, *48*(7), 943–963.

[CR12] González García, A. (2016). Factores de riesgo en el ciberacoso: revisión sistemática a partir del modelo del triple riesgo delictivo (TRD). In J. M. Tamarit (Eds.), *Ciberdelincuencia y cibervictimización. Revista de Internet, Derecho y Política*, 22, 73–92.

[CR13] González García, A., & Campoy Torrente, P. (2018). Ciberacoso y cyberbullying: Diferenciación en función de los precipitadores situacionales. *Revista Española De Investigación Criminológica*, *16*, 1–31.

[CR14] Gordon, K. R. (2018). High school students ’ perceptions of school climate in relation to discipline history and discipline approach, 1–134. https://doi.org/https://scholarworks.umass.edu/dissertations_2/1240

[CR15] Grasmick, H. G., Tittle, C. R., Bursik, R. J., & Arneklev, B. J. (1993). Testing the general theory of crime. *Journal of Research in Crime and Delinquency*, *30*(1), 5–29. 10.1177/0022427893030001002

[CR16] Hoeben, E. M., & Weerman, F. M. (2016). Why is involvement in unstructured socializing related to adolescent delinquency? *Criminology*, *54*(2), 242–281. 10.1111/1745-9125.12105

[CR48] Holt, T. J., Burruss, G. W., & Bossler, A. M. (2010). Social learning and cyber-deviance: Examining the importance of a full social learning model in the virtual world. Journal of Crime and Justice, 33(2), 31-61. 10.1080/0735648X.2010.9721287

[CR17] Holt, T. J., Cale, J., Brewer, R., & Goldsmith, A. (2021). Assessing the Role of Opportunity and Low Self-Control in Juvenile Hacking. *Crime & Delinquency,**67*(5), 662–688.

[CR18] Holt, T., Bossler, A. M., & May, D. C. (2012). Low self-control, deviant peer associations, and juvenile Cyberdeviance. *American journal of Criminal Justice*, *37*, 378–395. 10.1007/s12103-011-9117-3

[CR19] Hu, Q., Xu, Z., & Yayla, A. A. (2013). Why college students commit computer hacks: Insights from a cross culture analysis. *PACIS 2013 Proceedings*, 104. http://aisel.aisnet.org/pacis2013/104

[CR20] Janssen, H. J., Weerman, F. M., & Eichelsheim, V. I. (2017). Parenting as a protective factor against criminogenic settings? Interaction effects between three aspects of parenting and unstructured socializing in disordered areas. *Journal of Research in Crime and Delinquency,**54*(2), 181–207. 10.1177/0022427816664561

[CR21] Lee, B. H. (2018). Explaining Cyber Deviance among School-Aged Youth. *Child Ind Res*, *11*, 563–584. 10.1007/s12187-017-9450-2

[CR22] Lemmens, J. S., Valkenburg, P. M., & Gentile, D. A. (2015). The internet gaming disorder scale. *Psychological Assessment,**27*(2), 567–582. 10.1037/pas0000062 25558970 10.1037/pas0000062

[CR23] Lemmens, J. S., Valkenburg, P. M., & Peter, J. (2011). Psychosocial causes and consequences of pathological gaming. *Computers in Human Behavior,**27*(1), 144–152. 10.1016/j.chb.2010.07.015

[CR24] Leukfeldt, E. R., & Bekkers, L. M. J. (2026). From curiosity to crime: Exploring pathways into criminal hacking. *Computers in Human Behavior,**181*, Article 108996. 10.1016/j.chb.2026.108996

[CR25] Loggen, J., Moneva, A., & Leukfeldt, R. (2024). Pathways into, desistance from, and risk factors related to cyber-dependent crime: A systematic narrative review. *Victims & Offenders*. 10.1080/15564886.2024.2370295

[CR26] Maras, K., & Greenwood, J. (2024). Cyber offending in adolescence: The role of knowledge and experiences online on propensity to engage in unauthorised access. *Computers in Human Behavior Reports,**16*, Article 100492. 10.1016/j.chbr.2024.100492

[CR27] Marcum, C. D., Higgins, G. E., Ricketts, M. L., & Wolfe, S. C. (2014). Hacking in high school: Cybercrime perpetration by juveniles. *Deviant Behavior,**35*(7), 581–591. 10.1080/01639625.2013.867721

[CR28] Nagin, S. D. (2013). Deterrence in the 21st Century: A Review of the Evidence. *Crime and Justice*, *42*(1), 199–263.

[CR29] Pérez Ramírez, M. (2012). Riesgos personales, sociales y ambientales en la explicación del comportamiento antisocial: estudio empírico sobre el Modelo del Triple Riesgo Delictivo. Tesis doctoral no publicada. Departamento de Personalidad, Evaluación y Tratamiento. Psicológico, Facultad de Psicología, Universidad de Barcelona.

[CR30] Phillips, K., Davidson, J. C., Farr, R. R., Burkhardt, C., Caneppele, S., & Aiken, M. P. (2022). Conceptualizing cybercrime: Definitions, typologies and taxonomies. *Forensic Sciences,**2*(2), 379–398. 10.3390/forensicsci2020028

[CR31] Redondo, S. (2008). Individuos, Sociedades y Oportunidades en la explicación y prevención del delito: Modelo del Triple Riesgo Delictivo (TRD). *Revista Española de Investigación Criminológica*. 10.46381/reic.v6i0.34

[CR32] Redondo, S. (2015). *El origen de los delitos*. Tirant lo Blanch.

[CR33] Redondo, S., & Martínez, A. (2014). Inventario de Riesgos Individuales y Sociales (IRIS_R): un nuevo instrumento para evaluar factores de riesgo en consonancia con la estructura del Modelo TRD. *X Congreso Español de Criminología*, 50–51.

[CR34] Rogers, M., Seigfried, K., & Tidke, K. (2006a). Self-reported computer criminal behaviour: A psychological analysis. *Digital Investigation*, *3*, 116–120. 10.1016/j.diin.2006.06.002

[CR35] Rogers, M., Smoak, N. D., & Liu, J. (2006b). Self-reported deviant computer behaviour: A big-5, moral choice, and manipulative exploitive behaviour analysis. *Deviant Behavior*, *27*, 245–268. 10.1080/01639620600605333

[CR36] Rokven, J. J., Weijters, G., Beerthuizen, M., & van der Laan, A. M. (2018). Juvenile Delnquency in the Virtual World: similarities an differences between cyber-enabled, cyber-dependent and offline delinquents in the Netherlands. *International Journal of Cyber Criminology*, *12*(1). 10.5281/zenodo.1467690

[CR37] Seigfried-Spellar, K. C., & Treadway, K. N. (2014). Differentiating hackers, identity thieves, cyberbullies, and virus writers by college major and individual differences. *Deviant Behavior*, *35*(10), 782–803.

[CR38] Seigfried-Spellar, K. C., O’Quinn, C. L., & Treadway, K. N. (2015). Assessing the relationship between autistic traits and cyberdeviancy in a sample of college students. *Behaviour and Information Technology*, *34*(5), 533–542.

[CR39] Steinmetz, K. F. (2015). Craft(y)ness: An ethnographic study of hacking. *The British Journal of Criminology*, *55*(1), 125–145. 10.1093/bjc/azu061

[CR40] Udris, R. (2016). Cyber deviance among adolescents and the role of family, school, and neighborhood: A cross-national study. *International Journal of Cyber Criminology*, *10*(2), 127–146.

[CR41] Virgara, J. L., & Whitten, T. (2023). A systematic literature review of the longitudinal risk factors associated with juvenile cyber-deviance. *Computers in Human Behavior,**141*(2), Article 107613. 10.1016/j.chb.2022.107613

[CR42] Weulen Kranenbarg, M., Ruiter, S., & Van Gelder, J. L. (2021). Do cyber-birds flock together? Comparing deviance among social network members of cyber-dependent and traditional offenders. *European Journal of Criminology*, *18*(3), 386–406. 10.1177/1477370819849677

[CR43] Weulen Kranenbarg, M., Ruiter, S., van Gelder, J.-L., & Bernasco, W. (2018). Cyber-offending and traditional offending over the life-course: An empirical comparison. *Journal of Developmental and Life-Course Criminology,**4*(3), 343–364. 10.1007/s40865-018-0087-8 30956940 10.1007/s40865-018-0087-8 PMC6428311

[CR44] Weulen Kranenbarg, M., Van der Toolen, Y., & Weerman, F. (2022). *Understanding cybercriminal behaviour among young people: Results from a longitudinal network study among a relatively high-risk sample.* https://research.vu.nl/files/149641924/PUBLISHED_Understanding_cybercriminal_behaviour_among_young_people.pdf

[CR49] Wissink, I. B., Standaert, J. C. A., Stams, G. J. J. M., Asscher, J. J., & Assink, M. (2023). Risk factors for juvenile cybercrime: A meta-analytic review. Aggression and Violent Behavior, 70, 101836. 10.1016/j.avb.2023.101836

[CR45] Xu, Z., Hu, Q., & Zhang, C. (2013). Why computer talents become computer hackers. *Communications of the ACM,**56*(4), 64–74.

[CR46] Yar, M. (2005). Computer Hacking: Just another case of juvenile delinquency? *The Howard Journal*, *44*(4), 387–399. 10.1111/j.1468-2311.2005.00383.x

[CR47] Zullig, K. J., Koopman, T. M., Patton, J. M., & Ubbes, V. A. (2010). School climate: Historical review, instrument development, and school assessment. *Journal of Psychoeducational Assessment*, *28*(2), 139–152. 10.1177/0734282909344205

